# *Didiclis pseudobisulcata* (Selaginellaceae), a new species of the Bisulcata group from northern Thailand and southwest China

**DOI:** 10.3897/phytokeys.278.204624

**Published:** 2026-08-25

**Authors:** Sheng-Rong Gong, Yu-Ping Hu, Rossarin Pollawatn, Sahut Chantanaorrapint, Wen Wang, Liang Zhang, Xin-Mao Zhou

**Affiliations:** 1 School of Ecology and Environmental Science, Yunnan University, Kunming, 650500, Yunnan, China School of Ecology and Environmental Science, Yunnan University Kunming China; 2 Department of Botany, Faculty of Science, Chulalongkorn University, Bangkok 10330, Thailand Department of Botany, Faculty of Science, Chulalongkorn University Bangkok Thailand; 3 PSU herbarium, Division of Biological Science, Faculty of Science, Prince of Songkla University, Hat Yai, Songkhla 90110, Thailand CAS Key Laboratory for Plant Diversity and Biogeography of East Asia, Kunming Institute of Botany, Chinese Academy of Sciences (CAS) Kunming China; 4 Yunnan Huanglianshan National Nature Reserve Management Bureau, Lvchun 662599, China PSU herbarium, Division of Biological Science, Faculty of Science, Prince of Songkla University Songkhla Thailand; 5 CAS Key Laboratory for Plant Diversity and Biogeography of East Asia, Kunming Institute of Botany, Chinese Academy of Sciences (CAS), Kunming 650201, Yunnan, China Yunnan Huanglianshan National Nature Reserve Management Bureau Lvchun China

**Keywords:** Asia, Lycopodioidoideae, megaspore and microspore, phylogeny

## Abstract

*Didiclis pseudobisulcata* (Lycopodioidoideae, Selaginellaceae), a new lycophyte species from northern Thailand and southwest China, is described and illustrated. Morphologically, it is similar to *D. bisulcata* but can be reliably distinguished by its dorsal leaves on branches with an arista often more than 3/4 of the leaf length, the densely ciliolate base of the dorsal sporophyll pteryx, and megaspores with subreticulate exospore ornamentation. Molecular phylogenetic analysis based on ITS and *rbcL* sequences supports this species as an independent clade. The ITS sequences revealed more than 42 nucleotide differences between the new species and other members of the *Bisulcata* group, providing clear molecular evidence for its separation from its closest relatives.

## Introduction

Selaginellaceae is an ancient lineage of lycophytes, with origins dating back approximately 400 million years (Klaus et al. 2017). As the most species-rich family of lycophytes, Selaginellaceae currently comprises more than 755 species worldwide and exhibits a wide distribution and considerable morphological diversity (Zhou and Zhang 2015, 2023; Zhou et al. 2025). Based on phylogenetic analyses using one chloroplast marker (*rbcL*), two low-copy nuclear genes (*pgiC* and *SQD1*), three ribosomal genes (18S, 5.8S, and 26S), and 10 common morphological characters, Zhou and Zhang (2023) proposed a new infrafamilial classification for Selaginellaceae. This classification recognizes seven subfamilies, 19 genera, and six sections within Selaginellaceae. In a subsequent update, Zhou et al. (2025) established the subfamily Mexiselaginelloideae, which contains only one genus, *Mexiselaginella* Li Bing Zhang & X.M.Zhou, endemic to Mexico. Currently, Selaginellaceae comprises eight subfamilies and 20 genera (Zhou et al. 2025).

*Didiclis* is the second-largest genus in the subfamily Lycopodioidoideae, comprising approximately 80 species and occurring mostly in Asia (Zhou and Zhang 2023). Within *Didiclis*, six well-supported groups—the *Braunii* group, the *Bisulcata* group, the *Delicatula* group, the *Pervillei* group, the *Siamensis* group, and the *Willdenowii* group—were identified based on morphology and phylogeny. Although species of *Didiclis* show high gross morphological diversity, they often have tuberculate or verrucate megaspore surfaces and blunt-spiny to lamellate or cristate microspore surfaces (Zhou et al. 2015; Zhou and Zhang 2023). This unique morphological combination is distinctly different from that of other genera in Selaginellaceae.

In *Didiclis*, the *Bisulcata* group is the only group that possesses extremely wide, complanate strobili with dimorphic sporophylls. Dimorphic sporophylls occur mainly in *Hypopterygiopsis* and a few species of *Selaginella* s.s. (Zhou and Zhang 2023). In addition to dimorphic strobili, obovate dorsal leaves, nearly smooth exospore surfaces, and verrucate microspores easily distinguish this group from other groups in *Didiclis* (Zhou and Zhang 2023). Recently, *D. chiangdaoensis* X. M. Zhou, Yu P. Hu & Chantanaorr., a species endemic to northern Thailand, was published (Hu et al. 2025). Approximately eight species have been documented in this group (Zhou and Zhang 2023; Hu et al. 2025).

In recent years, following numerous field trips in Southeast Asia and China, a large number of Selaginellaceae specimens were collected. A literature review, field observations, and morphological comparisons of the collections confirmed that some specimens exhibit morphological differences from all known species within the *Bisulcata* group of *Didiclis*. After detailed comparison with closely related taxa, these collections were confirmed to possess a unique combination of morphological characteristics that does not match any previously described species in the *Bisulcata* group. These materials are described and illustrated here as a new species.

## Materials and methods

### Morphological study

Materials were collected during field trips in China and Thailand from 2019 to 2024. Specimens were compared with those of the *Bisulcata* group deposited at CDBI, KUN, and PYU (Index Herbariorum 2026). Megaspores and microspores were coated with gold using a BAL-TEC SCD 005 Cool Sputter Coater (BAL-TEC AG, Liechtenstein) and scanned using a QUANTA 200 scanning electron microscope (SEM) (FEI Co., USA) at 25 kV at Yunnan University, Kunming, China. The morphological terminology of spores follows Tryon and Lugardon (1991), Korall and Taylor (2006), Zhou et al. (2015), and Zhou and Zhang (2023). Herbarium investigations of species in the *Bisulcata* group were conducted at CDBI, PYU, and KUN. In addition, online images were examined through the Chinese Virtual Herbarium (CVH, http://www.cvh.org.cn), the Global Biodiversity Information Facility (GBIF, https://www.gbif.org/), and JSTOR Global Plants (https://plants.jstor.org/).

### Taxonomic sampling

To explore the phylogenetic positions of these materials, eight samples from different locations in China and Thailand were included. In total, 32 samples representing 19 species of *Didiclis* were used in the phylogenetic analyses. Based on previous phylogenetic studies (e.g., Zhou et al. 2022, 2025; Zhou and Zhang 2023), two species of Lycopodioidoideae, *Lycopodioides nipponica* (Franch. & Sav.) Kuntze and *Valdespinoa douglasii* (Hook. & Grev.) Li Bing Zhang & X.M.Zhou, were selected as the outgroups.

### DNA sequencing and phylogenetic study

The TIANGEN Plant Genomic DNA Extraction Kit (TIANGEN Biotech, Beijing, China) was used to extract total genomic DNA from silica-dried material following the manufacturer’s protocols. One plastid gene (*rbcL*) and one nuclear marker (ITS) were amplified and sequenced. The polymerase chain reaction (PCR) conditions were as described by Zhou et al. (2016). After amplification, the fragments were purified using TIANquick Mini Purification Kit (TIANGEN Biotech, Beijing, China), and the purified PCR products were sequenced by Tsingke (Beijing, China).

The newly generated sequences were edited and assembled using Sequencher v. 4.1.4 (Gene Codes Corporation, Ann Arbor, Michigan). Sequences were aligned using MAFFT v. 7 (Katoh and Standley 2013), with manual adjustment in BioEdit (Hall 1999). The matrix used for the phylogenetic analysis consisted of 2,160 characters (ITS1 + 5.8S + ITS2: 883 sites; *rbcL*: 1,277 sites). The two markers were concatenated using PhyloSuite v. 1.2.2 (Zhang et al. 2020). The best-fit substitution model was selected using ModelFinder (Kalyaanamoorthy et al. 2017). Based on the Akaike information criterion (AIC; Akaike 1974), the TIM3+F+R2 model was selected as the optimal model for both maximum likelihood (ML) and Bayesian inference (BI) analyses of the datasets. Maximum likelihood bootstrapping was performed using 5,000 rapid bootstrap (BS) replicates, followed by a search for the best-scoring tree in a single run using IQ-TREE v. 2.1.2 (Nguyen et al. 2015). Bayesian inference was performed using MrBayes v. 3.1.2 (Huelsenbeck and Ronquist 2001), with two runs, each comprising four Markov chain Monte Carlo chains. Each run began with a random tree, and one tree was sampled every 1,000 generations for 10,000,000 generations. The resulting phylogenetic trees were visualized and edited in FigTree v. 1.4.3 (Rambaut 2017).

## Results

A total of 32 sequences were newly generated for this study (Appendix). Comparison of the trees resulting from ML analyses of the individual markers (plastid *rbcL* and nuclear ITS) and the combined dataset (*rbcL* + ITS) did not reveal any well-supported conflicts. The concatenated alignment of 19 taxa was 2,160 characters long, of which 300 characters were parsimony-informative and 1,860 characters were parsimony-uninformative.

**Figure 1.**
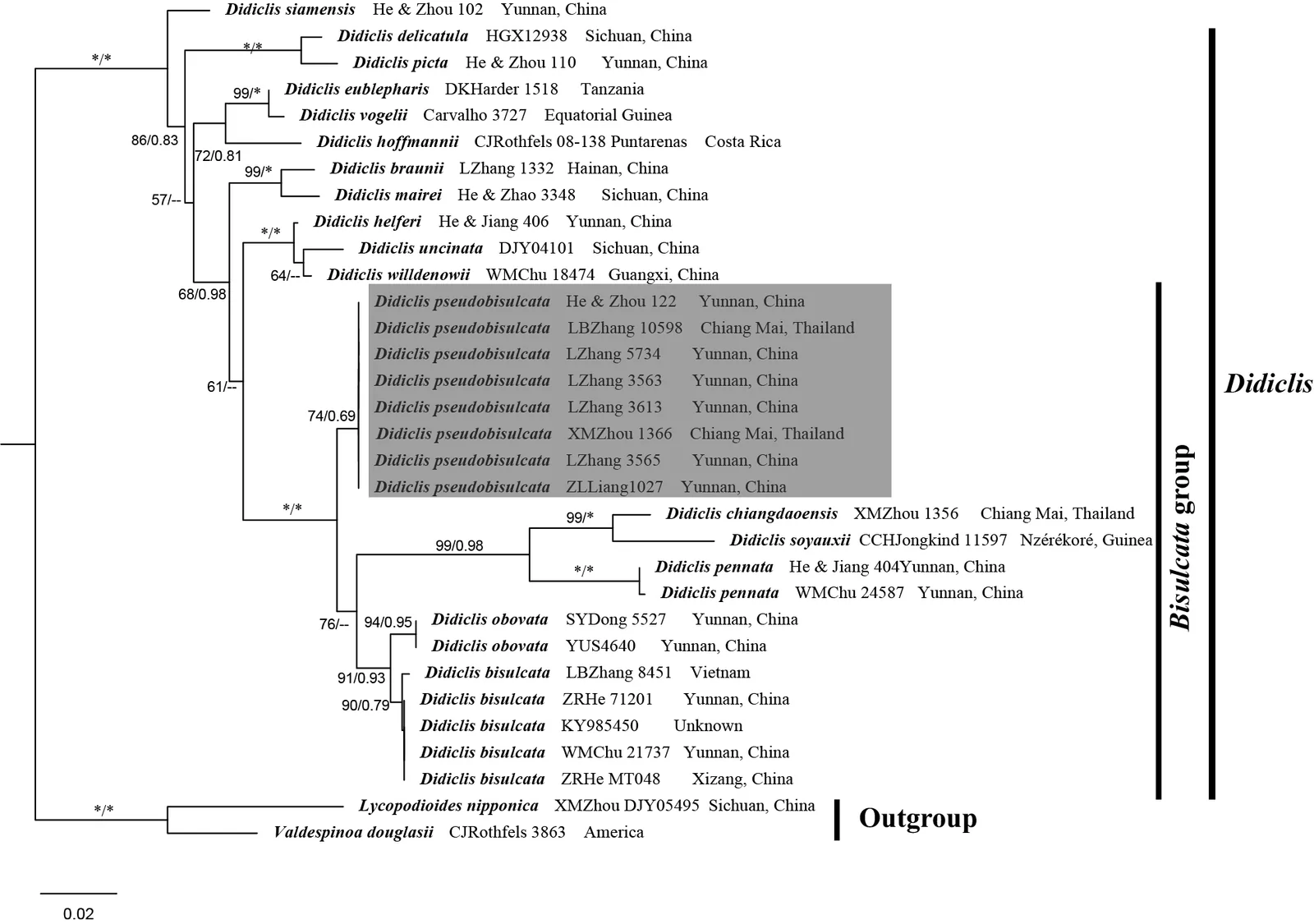
Maximum likelihood (ML) phylogenetic tree of *Didiclis pseudobisulcata* and its allies in the *Bisulcata* group based on the combined dataset of plastid *rbcL* and nuclear ITS sequences. Maximum likelihood bootstrap support (ML-BS) and Bayesian inference posterior probability (BI-PP) values are shown on the branches. A dash (--) indicates either ML-BS < 50 or BI-PP < 0.50; an asterisk (*) indicates ML-BS = 100 or BI-PP = 1.00; omitted support values indicate both ML-BS < 50 and BI-PP < 0.50.

Phylogenetic analyses based on ML and BI yielded slightly different topologies. In the ML tree, all eight accessions of the new species *Didiclis pseudobisulcata* formed an independent clade (ML-BS = 74; Fig. 1), which was resolved as sister to a clade containing *D. chiangdaoensis*, *D. soyauxii*, *D. obovata*, *D. bisulcata*, and *D. pennata*, but with weak support. In the BI tree, the monophyly of *D. pseudobisulcata* was also recovered (BI-PP = 0.69; Fig. 1), but it was resolved as sister to the clade comprising *D. obovata* and *D. bisulcata*, with weak support.

Morphologically, *Didiclis pseudobisulcata* is most similar to *D. bisulcata* and *D. obovata*. However, this new species differs in having subreticulate megaspore surfaces. Its dorsal leaves are aristate, with the arista more than half of the leaf length. The dorsal sporophylls bear a sporophyll pteryx extending to 4/5 of the leaf length, with the base densely ciliolate. Further morphological comparisons among *D. bisulcata*, *D. obovata*, and *D. pseudobisulcata* are provided.

Sequence variation analysis across species indicated clear divergence in the nuclear ITS region between *Didiclis pseudobisulcata* and other close relatives within the *Bisulcata* group. Compared with its relatives, *D. bisulcata* and *D. obovata*, *D. pseudobisulcata* harbors a large number of interspecific variable sites in the ITS region, with 45 and 42 variable sites, respectively.

## Discussion

The new species has a wide distribution, ranging from northern Thailand (Chiang Mai) to southwestern China (Yunnan). Although no specimens have yet been recorded from adjacent countries such as Myanmar, Vietnam, and Laos, its presence in these regions cannot be ruled out and warrants further field surveys. In Thailand, the new species is found at Doi Chiang Dao, located in the Doi Chiang Dao Wildlife Sanctuary, where another unique species of the *Bisulcata* group, *Didiclis chiangdaoensis*, was recently described (Hu et al. 2025). However, *D. chiangdaoensis* is found at higher elevations (ca. 2,200 m). Yunnan Province currently harbors the highest species diversity of the *Bisulcata* group in China, with approximately half of all known species in this group distributed there, including *D. bisulcata*, *D. obovata*, *D. pennata*, and *D. pseudobisulcata* (Huang et al. 2022; Zhou and Zhang 2023).

Within the *Bisulcata* group, *Didiclis pseudobisulcata* is distinguished as the sole species bearing subreticulate ornamentation on its megaspores; in contrast, the exospore surfaces of the megaspores of all other members of the group are characterized as nearly smooth (Zhou et al. 2015; Huang et al. 2022; Zhou and Zhang 2023).

Previous studies have used the ITS region as a barcode to identify species of Selaginellaceae (Gu et al. 2013; Pongkawong et al. 2021) and as an important DNA region for the phylogenetic reconstruction of Selaginellaceae (Zhou and Zhang 2015, 2023; Zhang et al. 2021). In this study, remarkably low intraspecific variation was detected in *D. pseudobisulcata*, with only two variable sites identified across five individuals; by contrast, interspecific divergence between *D. pseudobisulcata* and its congeners was substantial, with more than 42 variable sites recovered in the ITS sequences. Consistent with this contrast, the results indicate that the ITS region represents a potentially ideal DNA barcode for species identification within the *Bisulcata* group.

### Taxonomic treatment

#### Didiclis pseudobisulcata

Taxon classificationPlantaeSelaginellalesSelaginellaceae

X.M.Zhou & Liang Zhang sp. nov.

DC0C5E64-92B9-5990-B6AE-91D594200D59

urn:lsid:ipni.org:names:77395659-1

Figs 2, 3, 4

##### ‌Type‌.

‌China‌ • Yunnan Province: Lvchun County, Huanglianshan Mountain Nature Reserve, 22.89857760, 102.29593794, elev. ca. 1,908 m, 20 October 2024, *Liang Zhang, Xin-Mao Zhou, Lu-Yao Jiang & Xu-Ke Han 5734* (holotype: PYU-02074725!; isotype: PYU-02074726!, PYU-02074727!, PYU-02074728!, KUN!).

**Figure 2.**
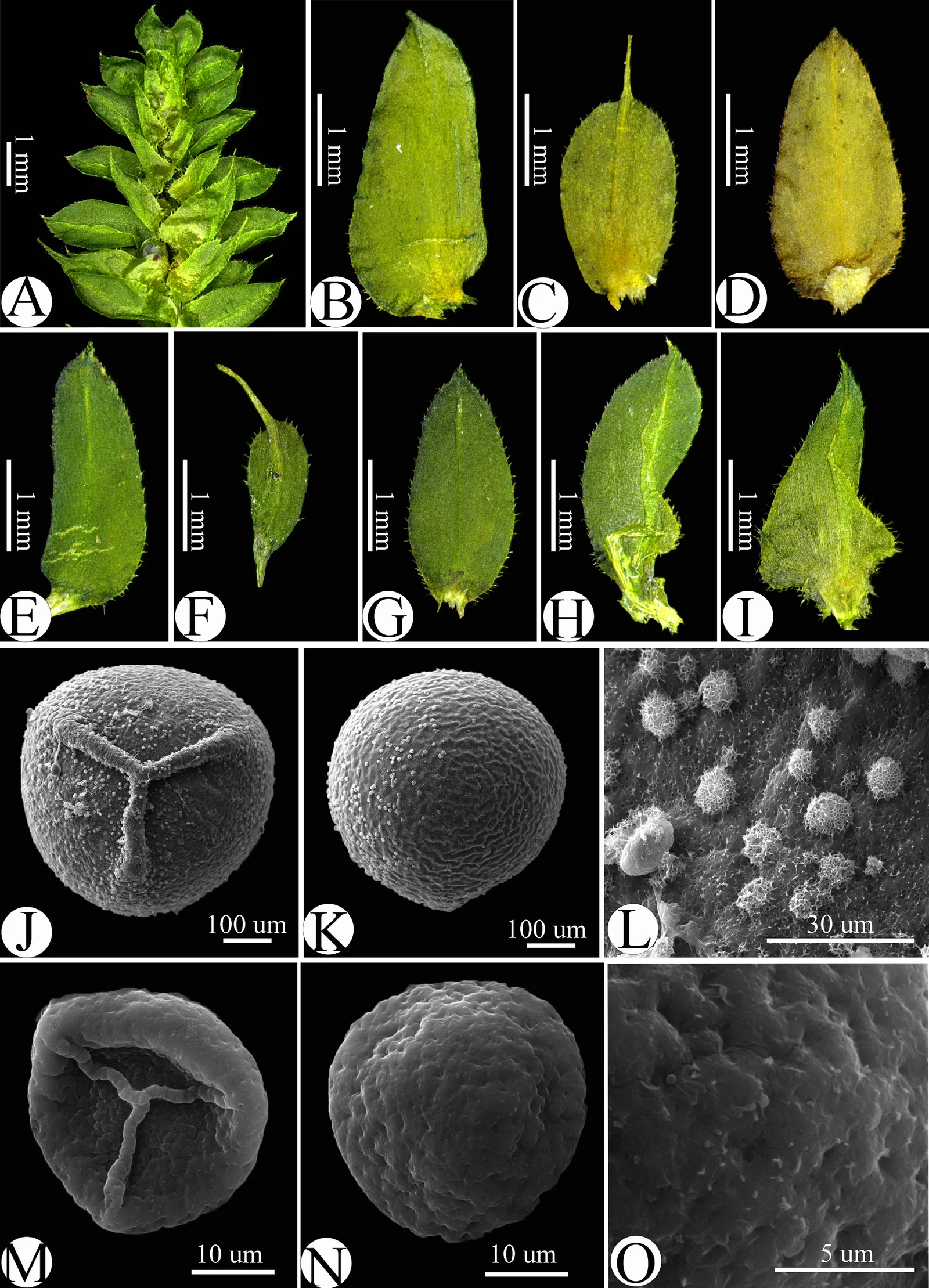
*Didiclis pseudobisulcata*. **A**. Ventral view of strobilus; **B**. Ventral leaf from the main stem; **C**. Dorsal leaf from the main stem; **D**. Axillary leaf from the main stem; **E**. Ventral leaf from a branch; **F**. Dorsal leaf from a branch; **G**. Axillary leaf from a branch; **H**. Dorsal sporophyll; **I**. Ventral sporophyll; **J**. Proximal surface of megaspore; **K**. Distal surface of megaspore; **L**. Ultrastructural detail of megaspore; **M**. Proximal surface of microspore; **N**. Distal surface of microspore; **O**. Ultrastructural detail of microspore (from voucher: *Liang Zhang, Xin-Mao Zhou, Lu-Yao Jiang, & Xu-Ke Han* 5734).

**Figure 3.**
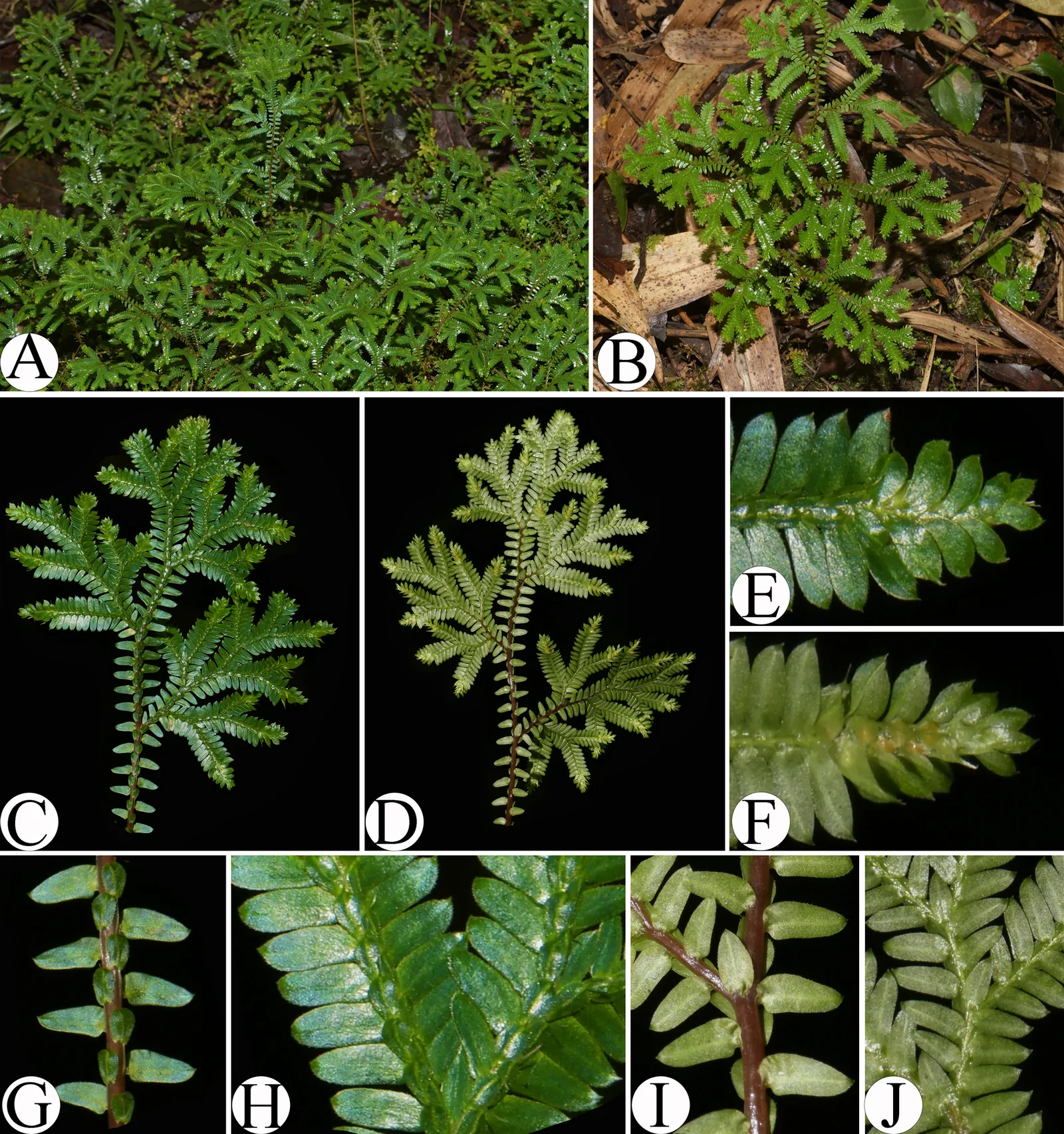
Field photographs of *Didiclis pseudobisulcata*. **A, B**. Plant in its natural habitat; **C**. Dorsal view of stems and branches with strobili; **D**. Ventral view of stems and branches with strobili; **E**. Dorsal view of strobilus; **F**. Ventral view of strobilus; **G**. Dorsal view of a portion of the main stem, showing ventral and dorsal leaves; **H**. Dorsal view of a portion of a branch, showing ventral and dorsal leaves; **I**. Ventral view of a portion of the main stem, showing axillary and ventral leaves; **J**. Ventral view of a portion of a branch, showing axillary and ventral leaves. (from voucher: *Liang Zhang, Xin-Mao Zhou, Lu-Yao Jiang, & Xu-Ke Han* 5734).

**Figure 4.**
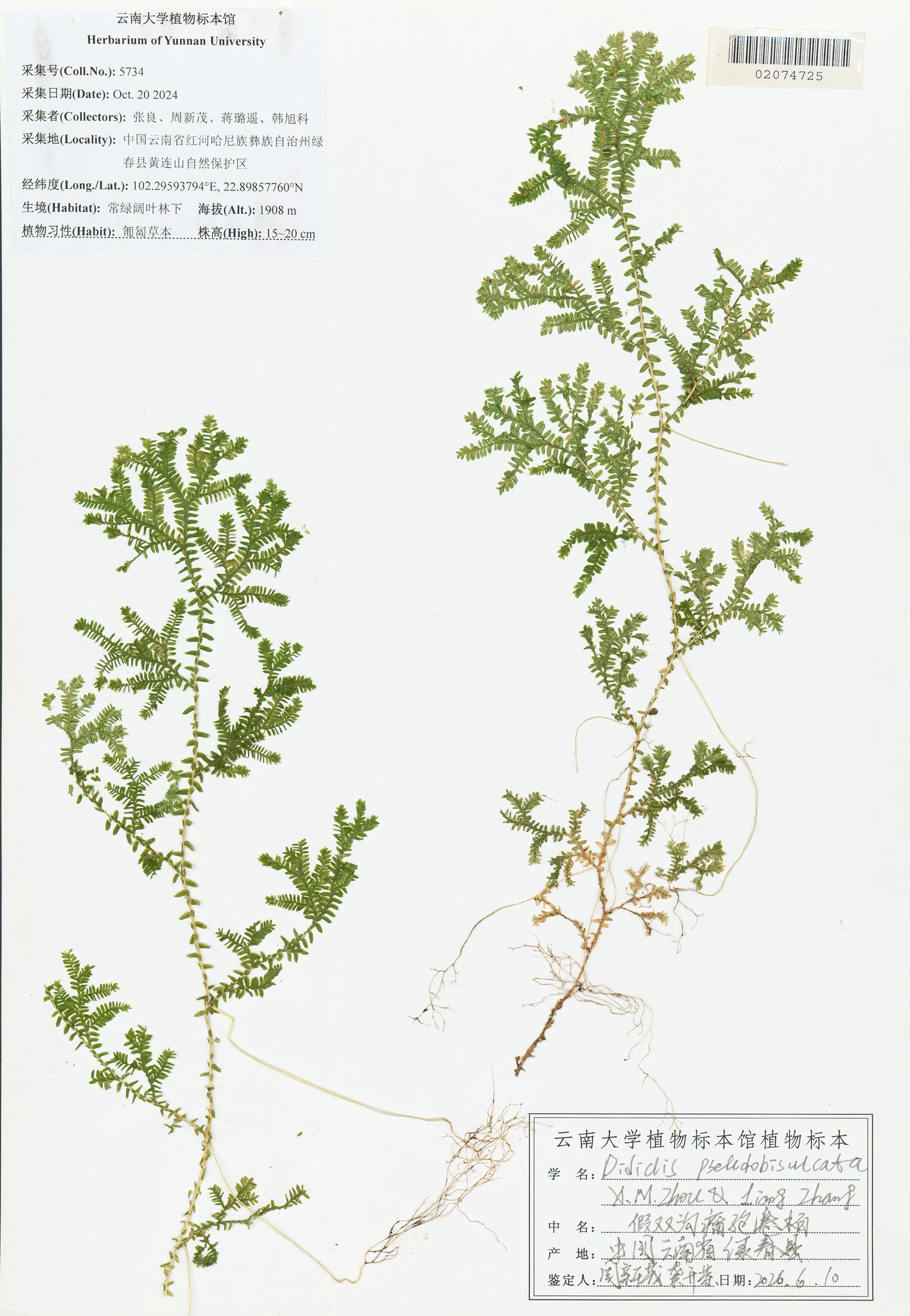
Holotype of *Didiclis pseudobisulcata* (*Liang Zhang, Xin-Mao Zhou, Lu-Yao Jiang, & Xu-Ke Han* 5734).

**Figure 5.**
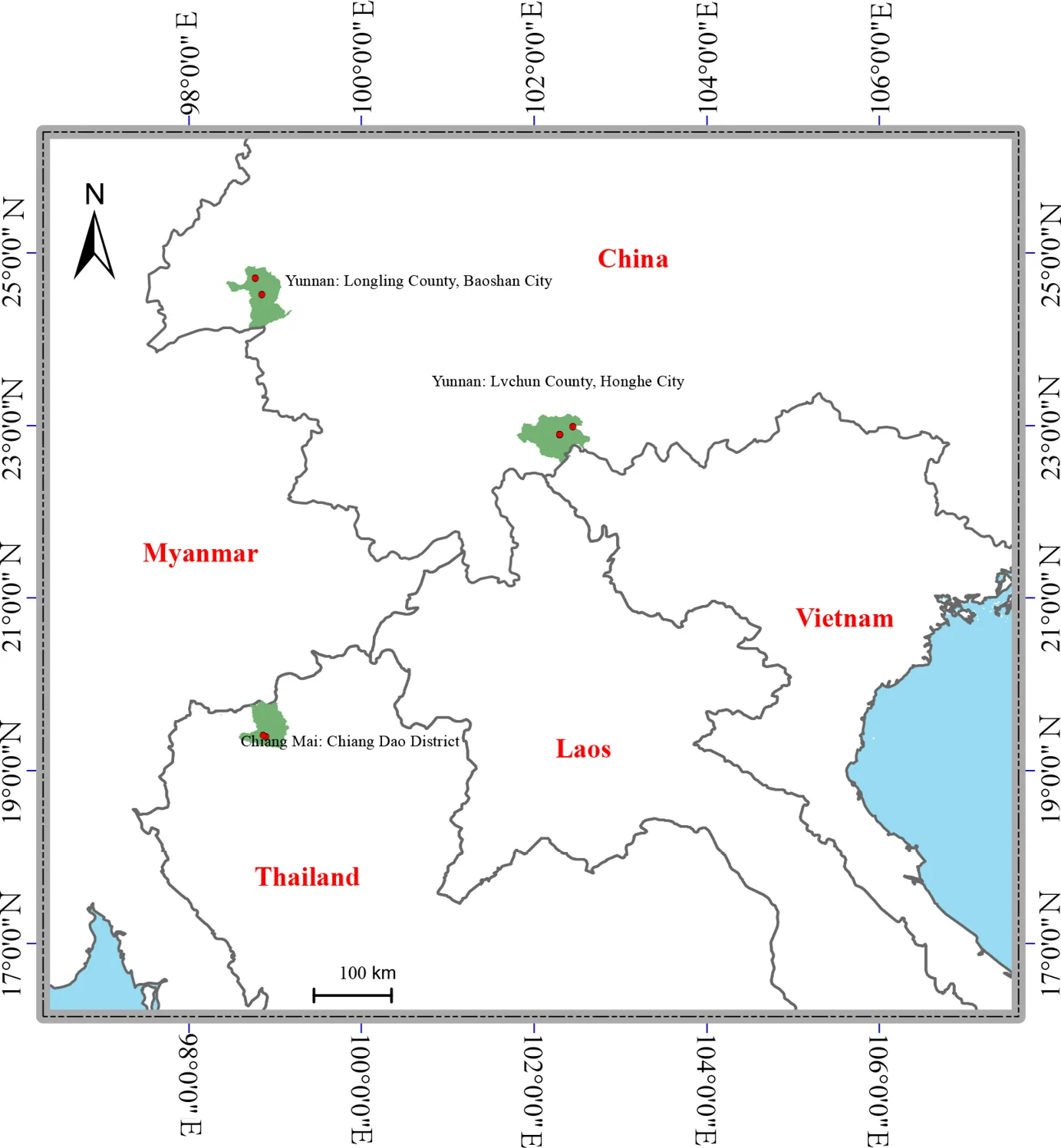
Geographical distribution of *Didiclis pseudobisulcata*.

**Table 1.** Morphological comparison among *Didiclis bisulcata*, *D. obovata*, and *D. pseudobisulcata*.

**Characters**	***D. bisulcata***	***D. obovata***	***D. pseudobisulcata***
Plants	20–40 cm	15–25 cm	15–20 cm
Stem diameter	1.1–2.1 mm	0.3–0.7 mm	0.6–0.9 mm
Apex of dorsal leaves on branches	Short-aristate, with arista 1/2–3/4 of leaf length	Short-apiculate	Long-aristate, with arista 1/2–4/5 of leaf length
Shape of axillary leaves	Ovate-lancolate	Elliptic to broadly elliptic	Ovate
Shape of ventral sporophylls	Ovate to ovate-lanceolate	Ovate-triangular	Ovate-lanceolate
Base margins of sporophyll-pteryx	Sparsely ciliolate	Subentire	Densely shortly ciliolate
Megaspore surface	Smooth, with oblate-spheroidal ornamentation	Smooth	Subreticulate, with regular white granules
Microspore surface	Low, partly fused tubercules	Irregularly verrucate, with microspines	Verrucate and slightly foveate

##### Diagnosis.

*Didiclis pseudobisulcata* is most similar to *D. bisulcata* in having creeping stems, more or less obovate dorsal leaves, strongly dorsiventrally flattened strobili, but can be distinguished by the combination of the following characters: dorsal leaves on branches with an arista often more than 3/4 of the leaf length (vs. less than 3/4 of the leaf length in *D. bisulcata*), base of the dorsal sporophyll pteryx densely ciliolate (vs. sparsely denticulate to ciliolate in *D. bisulcata*), and megaspores with subreticulate exospore ornamentation (vs. nearly smooth exospore in *D. bisulcata*).

##### Description.

***Plants*** terrestrial, evergreen, creeping, creeping stems 15–20 cm (Figs 3A, 3B, 4). ***Rhizophores*** borne intermittently on the main stem, on ventral side in axils of branches, 5–15 cm long, 0.19–0.40 mm in diam., stramineous, roots often forked (Fig. 4). ***Main stems*** pinnately branched from the base, 0.6–0.9 mm in diam. in lower part (Fig. 4), fresh purple but stramineous when dry, with one vascular bundle, subquadrangular, two sulcate, not zigzag, glabrous, primary leafy branches 7–10 pairs, 3–4 times pinnately branched, branchlets sparse to dense, adjacent primary branches on main stem 1.5–3.0 cm apart, leafy portion of main stem including leaves 5–8 mm wide at middle (Fig. 4), ultimate branches 3–5 mm wide including leaves (Figs 3I, 4). ***Sterile leaves*** not white-margined, margins shortly and slightly densely ciliolate (Fig. 2B–G). ***Axillary leaves*** on main stem slightly larger than those on lateral branches, symmetrical, ovate, 2.8–3.1 × 1.4–1.6 mm, apex acute, base obtuse, margins with very shortly and densely ciliolate and denticulate (Figs 2D, 3I, 3J); those on branches base obtuse to round, 1.7–2.9 × 1.0–1.4 mm (Fig. 2G). ***Dorsal leaves*** distant on stem, asymmetrical, those on main stems larger than those on branches (Figs 2C, 3G, 4); dorsal leaves on branches 2.2–3.5 × 0.9–1.6 mm, obliquely elliptic, overlapping each other at base, base obliquely cuneate, apex aristate, with arista 1/2–4/5 of leaf length, 0.9–1.6 mm (Figs 2F, 3H). ***Ventral leaves*** asymmetrical, those on main stem larger than those on branches (Figs 2B, 3I, 4); ventral leaves on branches obliquely ovate, distance between lateral leaves on branches near main stem 0.5–1.0 mm, ventral leaves denser towards branch tips, 3.3–4.8 × 1.5–2.0 mm, base obliquely cuneate, apex acute, acroscopic base slightly enlarged, rounded, shortly ciliolate or denticulate (Figs 2E, 3H, 3J, 4). ***Strobili solitary***, terminal, complanate, compact, resupinate, 5.6–6.4 mm (Figs 2A, 3E, 3F, 4); sporophylls strongly dimorphic; dorsal sporophylls oblong-falcate, not carinate on back, apex acute to mucronate, sporophyll-pteryx not reaching the leaf apex, extending to 4/5 of the leaf length, densely shortly ciliate at base (Fig. 2H); ventral sporophylls ovate-lanceolate, apex acuminate, base slightly enlarged, broadly cuneate, basal margins entire, densely ciliate, middle part sparsely ciliate; apical margins densely denticulate or shortly ciliate, sporophyll-pteryx extending to 2/3 of the sporophyll length, densely ciliate, indistinctly‌ carinate (Fig. 2I); megasporangia in basal portion on lower side of strobili. ***Microspores*** yellowish orange, hemispherical, 26.3–34.9 μm, laesurae extend 4/5 of the distance or extend to the equator, surface with indistinct verrucate ornamentation by slightly foveate and sparse small projections (Fig. 2M–O). ***Megaspores*** white or brown, globose, 516.9–615.2 μm, laesurae extend nearly 3/4 of the distance to the equator, surface subreticulate, covered with regular white granules composed by honeycombed structure (Fig. 2J–L).

##### Geographical distribution.

This species is currently known only from southwestern China (Yunnan) and northern Thailand (Chiang Mai).

##### Habitat.

This species grows beneath evergreen broad-leaved forests at elevations of 1,800–2,100 m in tropical regions.

##### Etymology.

The specific epithet “pseudobisulcata” derives from the Latin prefix *pseudo-* (meaning “false”) and the specific epithet bisulcata, referring to the strong morphological similarity between the new species and *Didiclis bisulcata*.

##### Additional specimens examined.

**China**. • **Yunnan Province**: Baoshan City, Longling County, Damo Mountain, by streams under broad-leaved forests, elev. ca. 2,100 m, 18 October 2019, *Zhen-Long Liang & Xue-Ping Fan 1027* (CDBI, KUN); Baoshan City, Xiaoheishan Nature Reserve, *Xin-Mao Zhou* & *Zhao-Rong He* 122 (PYU); Honghe City, Lvchun County, Daxing Town, Niubo Reservoir, elev. 1,810 m, 28 June 2020, *Liang Zhang, Xiang-Li Yue, Zi-Yu Ye, Jing Zhao, & Jian-Jun Yang 3613* (KUN, PYU); Sanmeng Township, elev. ca. 1,930 m, 8 June 2020, *Liang Zhang, Xiang-Li Yue, Zi-Yu Ye, Jing Zhao & Jian-Jun Yang 3563* and 3565 (KUN, PYU). **Thailand**. • **Chiang Mai Province**: Chiang Dao District, Doi Chiang Dao Wildlife Sanctuary, on the way to Kewlongtai, elev. ca. 2,000 m, 07 September 2019, *Li-Bing Zhang, Rossarin Pallawtn, Liang Zhang & Puttamon Nai* 10598 (KUN, CDBI, BCU, MO, PYU, XTBG), on the way to Doi Chiang Dao Summit elev. ca. 2,100 m, 08 October 2019, *Xin-Mao Zhou, Sahut Chantanaorrapint & Orawanya Suwanmala* 1366 (PSU, PYU).

## Supplementary Material

XML Treatment for Didiclis pseudobisulcata

## Appendix 1

***Didiclis bisulcata* (Spring) Li Bing Zhang & X.M. Zhou**, (1) He & al. 71201 (PYU), China (Yunnan): rbcL KT161406 (Zhou *et al*. 2016), ITS KT161675 (Zhou *et al*. 2016); (2) L.-B.Zhang *et al*. 8451 (CDBI, PYU), Vietnam (Lam Dong): rbcL PZ784112 (this study), ITS PZ778549 (this study); (3) Unknown: rbcL KY985450 (Klaus et al. 2017), ITS —; (4) W.-M.Chu *et al*. 21737 (PYU), China (Yunnan): rbcL KT161408 (Zhou *et al*. 2016), ITS —. ***Didiclis braunii* (Baker) Li Bing Zhang & X.M. Zhou**, Zhang 1332 (PYU, CDBI), China (Hainan): rbcL KT161420 (Zhou *et al*. 2016), ITS KT161686 (Zhou *et al*. 2016). ***Didiclis chiangdaoensis* Xin Mao Zhou, Y.P.Hu & Chantanaorr**, X.-M.Zhou1356 (PYU), Thailand (Chiang Mai): rbcL PZ784111 (this study), ITS PZ778551 (this study). ***Didiclis delicatula* (Desv. ex Poir.) Li Bing Zhang & X.M. Zhou**, Ju & Deng HGX12938 (CDBI), China (Sichuan): rbcL KT161440 (Zhou *et al*. 2016), ITS KT161700 (Zhou *et al*. 2016). ***Didiclis eublepharis* (A. Braun ex Hieron.) Li Bing Zhang & X.M. Zhou**, D.K.Harder *et al*. 1518 (L), Tanzania: rbcL KY023021 (Weststrand and Korall 2016), ITS —. ***Didiclis helferi* (Warb.) Li Bing Zhang & X.M. Zhou**, Z.-R.He & L.-J.Jiang 406 (PYU, CDBI), China (Yunnan): rbcL KT161470 (Zhou *et al*. 2016), ITS KT161723 (Zhou *et al*. 2016). ***Didiclis hoffmannii* (Hieron.) Li Bing Zhang & X.M. Zhou**, Rothfels & al. 08-138 (DUKE), Costa Rica (Puntarenas): rbcL KT161484 (Zhou *et al*. 2016), ITS KT161736 (Zhou *et al*. 2016). ***Didiclis mairei* (H. Lev.) Li Bing Zhang & X.M. Zhou**, He & Zhao 3348 (CTC) China (Sichuan): rbcL KT161516 (Zhou *et al*. 2016), ITS KT161763 (Zhou *et al*. 2016). ***Didiclis obovata* (S.Y. Dong) Li Bing Zhang & X.M. Zhou**, (1) X.-M.Zhou & J.-J.Yang YUS 4640 (PYU), China (Yunnan): rbcL PZ784110 (this study), ITS PZ778550 (this study); (2) S.Y.Dong 5527 (IBSC, PE), China (Yunnan): rbcL MW316870 (Huang *et al*. 2022), ITS—. ***Didiclis pennata* (D. Don) Li Bing Zhang & X.M. Zhou** (1) Z.-R.He & L.-J.Jiang 404 (PYU, CDBI), China (Yunnan): rbcL KT161558, ITS KT161798; (2) W.-M.Chu *et al*. 24587 (PYU), China (Yunnan): rbcL KT161557, ITS KT161797. ***Didiclis picta* (Griff.) Li Bing Zhang & X.M. Zhou**, Z.-R.He & X.-M.Zhou 110 (PYU, CDBI), China (Yunnan): rbcL KT161561 (Zhou *et al*. 2016), ITS KT161801 (Zhou *et al*. 2016). ***Didiclis pseudobisulcata*** (1) L.Zhang *et al*. 3565 (KUN, PYU), China (Yunnan): rbcL PZ779014 (this study), ITS PZ748784 (this study); (2) L.Zhang *et al*. 3613 (KUN, PYU), China (Yunnan): rbcL PZ779011 (this study), ITS PZ748786 (this study); (3) L.Zhang *et al*. 3563 (KUN, PYU), China (Yunnan): rbcL PZ779007 (this study), ITS PZ748787 (this study); (4) L.Zhang *et al*. 5734 (KUN, PYU): China (Yunnan), rbcL PZ779013 (this study), ITS PZ748782 (this study); (5) X.-M.Zhou *et al*. 1366 (CDBI, PYU): Thailand (Chiang Mai), rbcL PZ779010 (this study), ITS PZ748783 (this study); (6) Z.-R.He & X.-M.Zhou 122 (PYU, CDBI), China (Yunnan): rbcL PZ779008 (this study), ITS PZ748781 (this study); (7) L.-B.Zhang *et al*. 10598 (CDBI, PYU), Thailand (Chiang Mai): rbcL PZ779009 (this study), ITS PZ748785 (this study); (8) Z.-L.Liang 1027 (PYU), China (Yunnan), rbcL PZ779012 (this study): ITS —. ***Didiclis siamensis* (Hieron.) Li Bing Zhang & X.M. Zhou**, Z.-R.He & X.-M.Zhou 102 (PYU, CDBI), China (Yunnan): rbcL KT161600, ITS KT161831. ***Didiclis soyauxii* (Hieron.) Li Bing Zhang & X.M. Zhou**, C.C.H.Jongkind *et al*. 11597 (MO), Guinea (Nzérékoré): rbcL PZ784113 (this study), ITS PZ778552 (this study). ***Didiclis uncinata* (Desv. ex Poir.) Li Bing Zhang & X.M. Zhou**, L.Zhang & X.-M.Zhou DJY04101 (CDBI), China (Sichuan): rbcL KT161626 (Zhou *et al*. 2016), ITS KT161852 (Zhou *et al*. 2016); ***Didiclis vogelii* (Spring) Li Bing Zhang & X.M. Zhou**, Carvalho 3727 (S), Equatorial Guinea (Bioko): rbcL KY023175 (Weststrand and Korall 2016), ITS —. ***Didiclis willdenowii* (Desv.) Li Bing Zhang & X.M. Zhou**, W.-M.Chu & al.18474 (PYU), China (Guangxi): rbcL KT161640 (Zhou *et al*. 2016), ITS KT161864 (Zhou *et al*. 2016); ***Lycopodioides nipponica* (Franch. & Sav.) Kuntze**, L.Zhang & X.-M.Zhou DJY05495 (CDBI), China (Sichuan): rbcL KT161541 (Zhou *et al*. 2016), ITS KT161783 (Zhou *et al*. 2016). ***Valdespinoa douglasii* (Hook. & Grev.) Li Bing Zhang & X.M.Zhou**, Rothfels & al. 3863 (DUKE), USA (Oregon): rbcL KT161446 (Zhou *et al*. 2016), ITS KT161870 (Zhou *et al*. 2016).

## Additional information

### Artificial Intelligence (AI) use

The authors accept full responsibility for the content of the manuscript, including the disclosure of any use of AI.

No AI tools were used in the preparation of this manuscript.

### Author ORCIDs

S.-R. Gong https://orcid.org/0009-0006-7231-1352

Y.-P. Hu https://orcid.org/0009-0007-9915-9108

R. Pollawatn https://orcid.org/0000-0002-4612-2383

S. Chantanaorrapint https://orcid.org/0000-0002-9739-0994

W. Wang https://orcid.org/0009-0001-5681-1246

L. Zhang https://orcid.org/0000-0003-3784-3135

X.-M. Zhou https://orcid.org/0000-0003-3555-7784

### Data availability

All data supporting the findings of this study are available in the main text.
